# The prospects of African yam bean: past and future importance

**DOI:** 10.1016/j.heliyon.2020.e05458

**Published:** 2020-11-11

**Authors:** Toyosi T. George, Anthony O. Obilana, Samson A. Oyeyinka

**Affiliations:** a Department of Food Science and Technology, Faculty of Applied Sciences, Cape Peninsula University of Technology P.O Box 1906 Bellville, 7535, Bellville Campus, Cape Town, South Africa; b Department of Biotechnology and Food Technology, University of Johannesburg, DoornfonteinCampus, Gauteng, South Africa

**Keywords:** Food science, Food technology, Nutrition, African yam bean, Fortification, Enrichment, Phytochemicals

## Abstract

African yam bean (AYB) is an underutilised legume indigenous to West and East Africa with nutritional content comparable to other commonly consumed legumes. The nutrient density of the crop makes it a viable food crop for ameliorating the challenges of malnutrition faced in many developing countries, via direct consumption or fortification and enrichment of less nutritious staples. This review summarises the current body of knowledge on the use of African yam bean as a viable enrichment and fortification crop. Proximate composition and nutritional quality of foods (breakfast diets, traditional foods, snacks and instant noodles) fortified, enriched and complemented with AYB were discussed. The phytochemical and antioxidant potential of the crop were also discussed. Future studies should focus more on awareness towards the deliberate commercialisation of the crop and elevation of its status into a widely - consumed food in all households across developing countries. Adequate experimental design for optimum quantity to be used in the enrichment and fortification of many staples to improve their acceptance among consumers should be put in place. Phytochemical extracts of the bean are also proposed for use in the development of functional foods to mitigate against many lifestyle diseases.

## 1 Introduction

African yam bean (*Sphenostylis stenocarpa*) is a hard-to-cook underexploited leguminous plant grown extensively in Western Africa (Enujiugha et al., 2012; Uchegbu, 2015), Eastern and Central Africa (Bhat and Karim, 2009). In many areas where it is cultivated, AYB is regarded as a security crop for fallow farmlands in the preparation for a new planting season and primarily consumed as staple crops. It is a perennial crop (Nwosu, 2013), commonly or mainly planted by local farmers for subsistence (Klu et al., 2000; Enujiugha et al., 2012). The plant when harvested gives an edible seed and tuber (Adeyeye et al., 1994; Ajibola and Olapade, 2016) which is widely consumed in the South-eastern part of Nigeria (Idowu, 2014) and other parts of West Africa. The plant has a large genetic variation (Klu et al., 2000) with new accessions investigated recently (Olasoji et al., 2011; Abioye, 2015; Ajibola and Olapade, 2016; Baiyeri et al., 2018). Though rich in nutrients, it is still largely underexploited (Idowu, 2015a, Idowu, 2015b; Uchegbu, 2015). Nutritionally, the seed is rich in protein with values ranging between 19 and 30% (Nwokolo, 1987a; Edem et al., 1990; Adeyeye et al., 1994; Klu et al., 2000; Nwosu, 2013; Ndidi et al., 2014; Abioye et al., 2015; Ade-Omowaye et al., 2015; Duodu and Apea-Bah, 2017; Anya and Ozung, 2019). The protein in AYB compares favourably with those in pigeon pea, chickpea, Bambara, and common bean. The bean is also rich in dietary fiber (Ndidi et al., 2014; Baiyeri et al., 2018; Anya and Ozung, 2019), carbohydrate (Adeyeye et al., 1994; Oshodi et al., 1997; Klu et al., 2000; Nwosu, 2013; Ndidi et al., 2014; Ajibola and Olapade, 2016) and important minerals such as calcium, iron, zinc, magnesium amongst others with values higher or comparable to soy and common bean (Adamu et al., 2015). Protein fractionation shows that albumin and globulin are the prevalent proteins present in AYB (Ajibola et al., 2016). The bean is rich in essential amino acids (Ade-Omowaye et al., 2015; Chinonyerem et al., 2017). Therefore, more attention needs to be given to this underexploited, cheap, and nutritionally important legume.

Because AYB is rich in protein and other nutrients, it has been used in the development of fortified and enriched foods, although both words were used interchangeably by some authors. Food fortification has been described as the inclusion of important micronutrients and vitamins not originally present or present in infinitesimal quantity within foods from other foods (Oyeyinka and Oyeyinka, 2018; Nwadi et al., 2020), whereas food enrichment involves the addition of nutrients (macro or micro alike) to foods to compensate for losses that may have occurred during processing (Nwadi et al., 2020). The bean has been used in the development of cookies and snacks (Idowu, 2014; Igbabul et al., 2015), an imitation yoghurt (Amakoromo et al., 2012), composite flour with rice and brown cowpea seeds (Iwe et al., 2016), breakfast meals (Babarinde et al., 2019), traditional snack food such as *Kokoro* (Idowu, 2015b), maize – AYB meal composite (Idowu, 2015a), AYB enriched *fufu* (Aniedu and Aniedu, 2014). Therefore, they can be used to fortify other foods low in protein to address protein malnutrition among the susceptible population. The bean has been touted for use in addressing the problem of food insecurity in Africa because they are indigenous (Klu et al., 2000; Abdulkareem et al., 2015; Abioye et al., 2015). Additionally, as a pulse, it can thrive under unfavourable climatic conditions, withstanding a prolonged period of drought and hence can become a viable source of plant protein with the looming problems of climate change. Hence, the many potentials inherent in the bean should be harnessed.

Aside from the high nutrient content, the bean has been reported to also be a source of phytochemicals and bioactive compounds that offer health benefits such as the mitigation of lifestyle diseases to consumers (de Vos et al., 2010; Ade-Omowaye et al., 2015; Uchegbu, 2015; Duodu and Apea-Bah, 2017; Soetan et al., 2018). Phytochemicals are secondary metabolite compounds found in various plants, fruits, and seeds in smaller quantities compared to primary metabolites (Larayetan et al., 2019). These compounds have been reported to help in decreasing the risk of diseases such as cardiovascular disorder and other degenerative diseases associated with an antioxidant imbalance in the body (Ade-Omowaye et al., 2015; Oboh et al., 2009; Oboh, 2006) caused by cellular damage induced by oxidative stress (Padhi et al., 2017). Flavonoids and phenolic acids are two important bioactive compounds that have been identified in AYB (Ade-Omowaye et al., 2015; Oboh et al., 2009; Oboh, 2006; Soetan et al., 2018). Therefore, the cooked seed can be an effective dietary inclusion among individuals that are diabetic, have cardiovascular disorders, and other lifestyle ailments. Additionally, because of the presence of fibre, some useful starch, and essential fatty acids, AYB is a good candidate for the development of new functional foods for consumer healthiness.

There are a few or no up-to-date reviews on the potential of African yam bean. Therefore, this review seeks to provide up-to-date information on the bean, the nutritional and proximate composition of foods enriched, fortified, and complimented with AYB, and phytochemical properties of African yam bean, limitation to its use, as well as further awareness as food, that can contribute to mitigating the problem of food security in Africa.

## 2 Proximate and nutritional composition of African yam bean

Different authors over the years have reported the proximate composition of African yam bean with varying values over the years (Table 2). Carbohydrate (49.88–63.51%) and protein (19.53–29.53%) are the major components of AYB, while other components such as ash (1.86–5.35%), fat (1.39–7.53%), and fibre (2.47–9.57%) are present in relatively small amounts. Several factors including different seed accession, planting location, agronomic practices as well as seasons of the year when the bean was planted may influence their proximate composition. Some authors have studied different variants of AYB and reported slight differences in protein content 22.33–25.78% (Adeyeye et al., 1994), 22.72–26.68% (Ajibola and Olapade, 2016) 21.84–23.41% (Baiyeri et al., 2018).

Other authors also reported varying protein content with different processing conditions. For instance, Ndidi et al. (2014) found a slight but significant reduction in the protein content of boiled and roasted AYB. Boiled AYB showed approximately a 9% reduction in protein compared to the roasted grain (approx. 6%). Nwosu (2013) also reported a slight increase (2.5 and 5% respectively) in the protein content of AYB after steeping for 24 and 48 h, and then germination at 96 h. The author attributed the increase to the synthesis of protease during germination (Nwosu, 2013). The results from both authors further reinforce the influence of different treatment on the protein content of the bean.

The presence of amino acids (8 essential and 9 non-essential) has been reported, and the most prevalent of them were; glutamic acid, aspartic acid, leucine, and lysine (Ade-Omowaye et al., 2015; Chinonyerem et al., 2017). Sulphur-containing amino acid - cysteine and methionine, were only present in minute quantity as in most other pulses (Ade-Omowaye et al., 2015). Furthermore, the amino acid composition of the bean varies among cultivars (Adeyeye, 1997). The presence of amino acids makes the bean an excellent fortifying candidate for many cereal-based diets that are deficient in protein, and as such can be used in addressing the problem of kwashiorkor and marasmus among infants.

The bean is low in lipid, the low-lipid content is expected since AYB is classified as a pulse. Pulses, unlike oil seeds, have very low fats usually less than 10% but may sometimes be slightly higher. AYB contains alpha-linolenic and linoleic acid (Ade-Omowaye et al., 2015). The oleic acid (34.40 g/kg) content was also reported by the same author and found to be higher than some other pulses. All of these are important in human nutrition as they are good for the general wellbeing of the human body but cannot be synthesized by the body and hence must be supplied via the diet. The low-fat content of AYB also makes their handling, processing, and storage easy (Abioye et al., 2015; Baiyeri et al., 2018). High fatty foods are prone to oxidative damage during processing and storage which results in decreased shelf-life and sensory quality.

**Figure 1:**
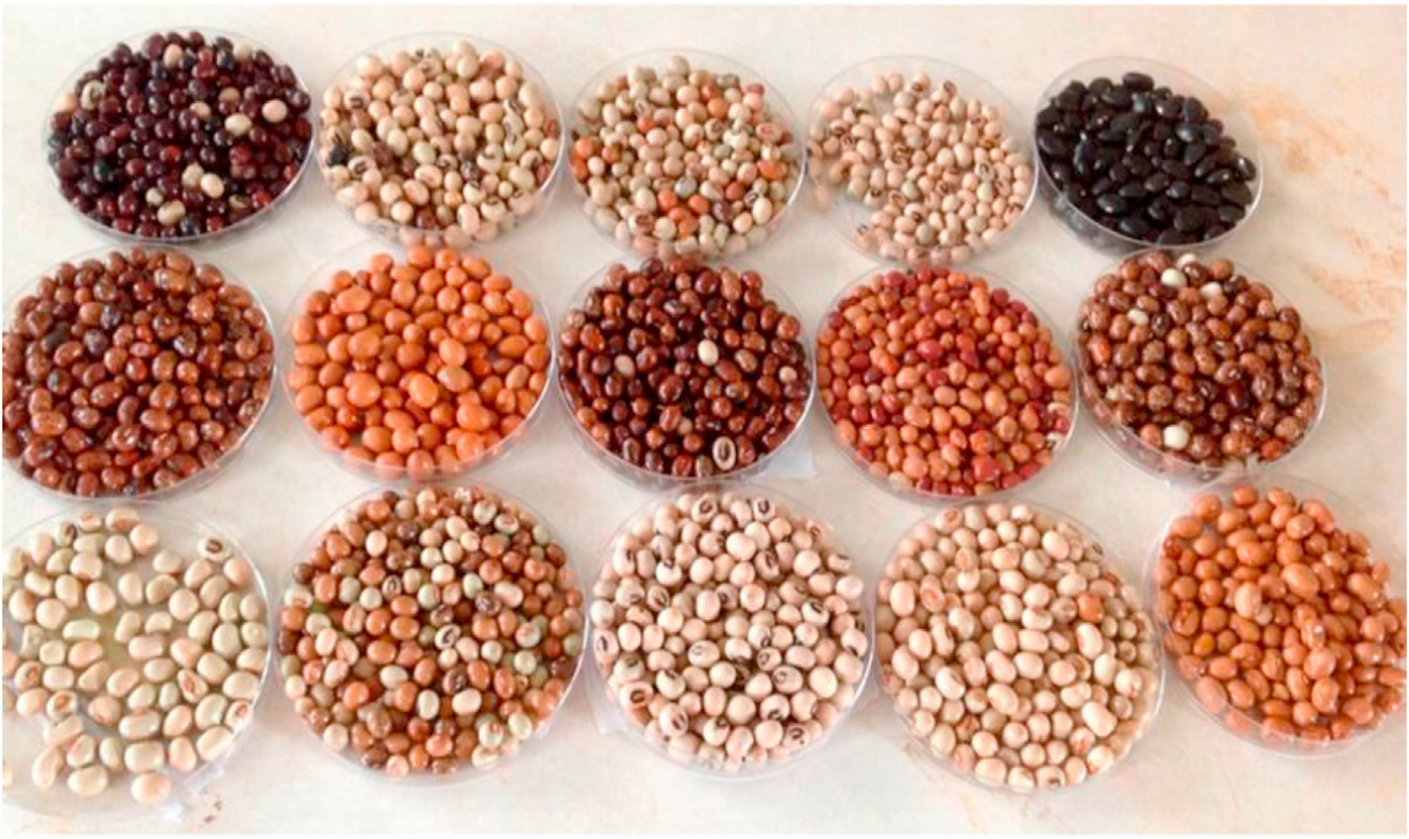
Variants of African yam bean seeds (Nnamani et al., 2017).

African yam bean contains an amount of carbohydrate (CHO) (49.88–63.51%), comparable to some other legumes (Table 2). Some authors have reported varying values for the CHO of AYB. Variation in CHO content as other chemical composition can be attributed to the variation in locations where they are sourced, genetic differences, processing conditions, agronomic and climatic conditions of where they are planted. This seems plausible since the grains come in different sizes shapes and colour (Figure 1).

The bulk of CHO in the bean is starch (28.08%) and this starch has slowly digestible properties, which are fit for the consumption of diabetic patient since they do not spike the glycemic index and blood glucose of individuals suffering from diabetes (Maphosa and Jideani, 2017; Baiyeri et al., 2018). Besides the starch content, Ade-Omowaye et al. (2015) reported that the bean is also a good source of non-starch polysaccharides (NPS) such as cellulose, hemicellulose and arabinose (Kumar et al., 2012). NPS lowers the risk posed by cardiovascular disorder, colorectal cancer, breast cancer, coronary heart diseases (CHD), laxative disorder, type 2 diabetes and other lifestyle disorder (Kumar et al., 2012).

African yam bean contains crude fibre content between 2.47 and 9.57% soluble and insoluble alike (Table 1). The presence of fibre in AYB makes them a potential source of important functional foods to consumers because fibre consumption lowers the incidence of lifestyle diseases among consumers. Additionally, with the growing consumer awareness on the need to consume healthy foods AYB can be a source of inexpensive, yet health-benefiting food commodity to consumers across all socio-economic statuses.

**Table 1:** Proximate composition of African yam bean from different authors in percentage (%).

Region of Purchase of Sample	Ash	Protein	CHO	Fibre	Fat	Moisture	References
Nsukka	4.55	19.62	NR	8.45	3.00	NR	(Nwokolo, 1987a)
Ekiti	2.60	20.51	50.24	6.09	12.20	8.36	(Adeyeye et al., 1994)
Owerri	3.23	23.24	58.05	5.31	1.64	9.43	(Nwosu, 2013)
Southern Kaduna	2.64	21.78	63.51	9.29	2.84	9.23	(Ndidi et al., 2014)
Southwest Nigeria	NR	19.53	NR	NR	1.39	12.04	(Ade-Omowaye et al., 2015)
Southwest Nigeria	4.30	24.96	59.85	6.26	2.70	1.93	(Adamu et al., 2015)
Enugu	3.66	27.66	49.88	9.57	4.29	4.94	(Ikpa, 2015)
IAR&T & NACGRAB	3.47	29.53	52.59	2.75	2.80	9.03	(Abioye, 2015)
IITA Ibadan	1.86	24.31	58.64	2.47	1.88	13.30	(Ajibola and Olapade, 2016)
IITA Ibadan	4.28	22.46	53.68	6.47	3.59	9.52	(Baiyeri et al., 2018)
Cross River	5.35	21.61	NR	7.00	5.12	NR	(Anya and Ozung, 2019)

NR: Not reported, CHO: Carbohydrate.

IITA: International Institute of Tropical Agriculture.

IAR&T: Institute of Agricultural Research & Training.

NACGRAB: National Centre for Genetic Resources and Biotechnology.

The bean is low in moisture (1.93–13.30%) with the exception of certain varieties, these values are within the recommended moisture value for safety (14.50%) (Abioye et al., 2015), although moisture content largely depends on postharvest drying, handling, and storage conditions. Some authors also reported the presence of important mineral compounds that are essential micronutrients in appreciable amount (Nwokolo, 1987a; Ibiyinka et al., 2014; Abioye et al., 2015; Duodu and Apea-Bah, 2017), although a high calcium content (48.33–85.00 mg) was reported by Abioye et al. (2015).

All these have shown the importance of this underexploited pulse and can be used in the development of novel food products as well as functional foods that are important for healthy living. The proximate composition data for AYB compares favourably with values reported for other common pulses (Table 2) such as cowpea (Klu et al., 2000; Preet and Punia, 2000; Henshaw, 2008), Bambara groundnut (Onimawo et al., 1998; Ijarotimi and Esho, 2009; Olanipekun et al., 2012; Ade-Omowaye et al., 2015), chickpea (Rincón et al., 1998; Iqbal et al., 2006; Nikolopoulou et al., 2006), common bean (Rathna Priya and Manickavasagan, 2020) and pigeon pea (Nwokolo, 1987b; Olalekan and Bosede, 2010; Adebowale and Maliki, 2011; Jeevarathinam and Chelladurai, 2020).

**Table 2:** Proximate composition of African Yam Bean in Comparison with some common and underutilized legumes (%).

Legume	Ash	Protein	Carbohydrate	Fat	Fibre	Moisture	References
African yam bean	1.86–5.35	19.53–29.53	49.88–63.51	1.39–7.53	2.47–9.57	1.93–13.30	(Nwokolo, 1987a; Adeyeye et al., 1994; Nwosu, 2013; Abioye et al., 2015; Adamu et al., 2015; Ade-Omowaye et al., 2015; Ikpa, 2015; Baiyeri et al., 2018; Anya and Ozung, 2019)
Cowpea	3.40–4.50	19.00–27.00	56.00–64.00	1.00–4.50	1.30–2.50	6.80–9.10	(Henshaw, 2008; Owolabi et al., 2012)
Soybean	4.40	35.00	35.00	17.00	NR	13.00	(Liu, 1997)
Bambara	2.50–3.50	17.00–25.00	53.00–69.00	6.50–8.50	1.80–14.00	NR	(Onimawo et al., 1998; Olanipekun et al., 2012; Ade-Omowaye et al., 2015; Manickavasagan and Saha, 2020)
Chickpea	3.20–3.90	19.00–25.00	41.1–47.4	4.50–6.00	17.40	7.20–7.40	(Iqbal et al., 2006; El-Adawy, 2002; Sofi et al., 2020)
Pigeon pea	3.50–4.05	19.39–22.30	57.16–57.60	1.70–3.24	1.50–5.56	NR	(Jeevarathinam and Chelladurai, 2020)

NR: Not reported.

## 3 Phytochemical composition of African yam bean

Pulses contain several biologically active compounds that possess health-promoting attributes against several lifestyle diseases (Maphosa and Jideani, 2017; Duodu and Apea-Bah, 2017). African yam bean is one of such, rich in bioactive such polyphenols and flavonoids that are important and beneficial for consumers to keep and maintain good living (Oboh, 2006; Oboh et al., 2009; Ade-Omowaye et al., 2015; Soetan et al., 2018). Polyphenols and flavonoids are compounds widely distributed in plants and they are present in seeds, tissues, stems, fruits, bark, and roots of most plants (de la Rosa et al., 2019). Awika and Duodu (2017) noted that they are usually present in the cell wall of many plants and possess defensive functions. These compounds have received great attention in recent times from researchers because of their potential in ameliorating the damage caused by oxidative stress in the human body (Anwar et al., 2007; Jideani and Diedericks, 2014; Awika and Duodu, 2017; Duodu and Apea-Bah, 2017), and have been suggested as possible replacements for synthetically manufactured antioxidants (Bhanja Dey et al., 2016). They exist in bound and free form in many plants (Govardhan Singh et al., 2013), and are originally present in plants for defence against predators, but have now found great importance in combatting life-threatening health problems in humans (Jideani and Diedericks, 2014).

Biologically active compounds and antioxidant activities in African yam bean have been reported by some authors (Table 3). The phenolic and flavonoid content, and antioxidant activity as well as the potential of AYB in scavenging free radicals may differ with different AYB varieties.

**Table 3:** Phytochemical and Antioxidant activity of treated and untreated African yam bean by different authors.

AYB samples	Phenolic content	Flavonoid	DPPH	FRAP	References
AYB seed	0.7 mg/g	NR	23.6%	0.05 OD_700_	(Oboh, 2006)
Fermented bean seed	0.47 mg/g	NR	90%	0.65 OD_700_	(Oboh et al., 2009)
Unfermented bean seed	0.33 mg/g	NR	84.10%	0.23 OD_700_	(Oboh et al., 2009)
Raw AYB seed	0.75–3.29 mg GAE/g	0.08–0.22 mg CE/g	0.07–0.09 μmol TE/g	NR	(Enujiugha et al., 2012)
Dried AYB seed	1.27–1.32 mg GAE/g	0.19–1.02 mg CE/g	0.06–0.09 μmol TE/g	NR	(Enujiugha et al., 2012)
Autoclaved AYB seed	1.25–1.98 mg GAE/g	0.14–0.17 mg CE/g	0.063–0.09 μmol TE/g	NR	(Enujiugha et al., 2012)
AYB seed	288.68 mg/100g	NR	NR	NR	(Ade-Omowaye et al., 2015)
Germinated AYB seed	117.08 mg/100g	68.31 mg/100g	48.92 μg/ml	NR	(Uchegbu, 2015)
Ungerminated AYB seed	98.27 mg/100g	51.64 mg/100g	31.11 μg/ml	NR	(Uchegbu, 2015)

NR: Not reported.

For example, varying total phenolic content (TPC) values have been reported in different studies, variations can be ascribed to differences in genetic makeup (Soetan et al., 2018), the influence of processing and treatments (Enujiugha et al., 2012; Oboh et al., 2009), as well as variation in extraction protocols (Enujiugha et al., 2012). Soetan et al. (2018), reported TPC values ranging from 0.63 – 1.99 mg GAE/ml for six different variants of AYB extracted with acetone and water. Uchegbu (2015) also reported TPC values of 117.08 and 98.27 μmol GAE/100 g for germinated and ungerminated samples of AYB. Oboh et al. (2009) reported TPC values of 0.47 and 0.33 mg/g for fermented and unfermented AYB respectively. These authors reported that fermented AYB samples have higher TPC values than unfermented samples, this is because fermentation enhances the bioavailability of phenolic compounds. The breakdown of cell walls of beans, hydrolysis of certain compounds such as antinutrients and high molecular weight polyphenols contributes to high phenolic content noticed in fermented samples (Hur et al., 2014). Enujiugha et al. (2012) also reported varying TPC values for raw (0.75–3.29 mg GAE/g), dried (1.27–1.32 mg GAE/g), and autoclaved (1.25–1.98 mg GAE/g) samples of AYB extracted using three solvents (80% ethanol, 80% acetone and acidified 70% acetone). Other authors reported varying TPC values for raw AYB samples. For instance, 0.7 mg/100g, and 288.68 mg/100g, reported by (Oboh, 2006), and Ade-Omowaye et al. (2015) respectively with variations likely due to the cultivar used. These values indicate the richness of this legume in phenolic compounds which are important compounds against free radicals in the human body that causes an imbalance in the antioxidant levels of the body and results in oxidative damage (Oboh, 2006). Hence, they can be consumed as prophylactic foods against diseases such as cancer, diabetes, high blood pressure, cardiovascular disease among other life-threatening diseases (Duodu and Apea-Bah, 2017; Maphosa and Jideani, 2017). The bean is also reported to be rich in total flavonoids, with the amount varying with the extraction solvent, variety of grain used, and the processing methods (Uchegbu, 2015; Enujiugha et al., 2012; Soetan et al., 2018). Germinated AYB reportedly showed significantly higher TFC (68.31 mg/100g) than the ungerminated bean (48.92 mg/100g) Uchegbu (2015). Enujiugha et al. (2012) found that extraction solvent influence the TFC of the grain with acidic 70% acetone having the best efficiency over 80% acetone and 70% ethanol. Raw AYB had lower TFC (0.08–0.22 mg CAE/g) compared to the dried (0.19–1.02 mg CAE/g) and autoclaved samples (0.14–0.17 mg CAE/g). More recently, Soetan et al. (2018) reported that acetone extracted more flavonoids and phenolic compounds for different variants of AYB than water. All these authors also noted that flavonoids in foods have antioxidant effects and are effective prophylactic and therapeutic compounds against several diseases.

A high phytochemical content is a good indicator of high antioxidant activities such as free radical scavenging potential (Enujiugha et al., 2012; Oboh et al., 2009; Soetan et al., 2018), reducing power (Oboh, 2006; Oboh et al., 2009; Soetan et al., 2018) and metal chelating effects (Soetan et al., 2018). Furthermore, Ibiyinka et al. (2014) stated that other compounds such as carotenoids, vitamins, and volatile oils present in AYB also contribute to its antioxidant activity and as such the consumption should be encouraged. The presence of these phytochemicals makes this food crop one that should be exploited and used in the production of functional foods. Inclusion into food would not only enrich or fortify foods but would also serve as a natural way of staying healthy for consumers. The phytochemical and antioxidant activities of AYB as reported by certain authors are detailed in Table 3.

## 4 The importance of African yam bean in food fortification and enrichment

African yam bean has been used in the fortification and enrichment of other less nutritious foods in the past. Food fortification involves the inclusion of micronutrients and vitamins that are not initially present or present in diminutive quantity within the foods, whereas food enrichment involves the addition of nutrients (macro or micro alike) to foods to compensate for losses that may have occurred during processing. Most authors still use both terms interchangeably. Fortification and enrichment of foods low in certain nutrients improve the nutritional status of developing countries where the cost of buying foods rich in animal protein is unaffordable (Bolarinwa et al., 2019). For effective fortification of food, fortifiers should be accessible and available so that its effect on the larger population can be felt (Oyeyinka and Oyeyinka, 2018). Additionally, the process of enhancing the nutritional quality of foods, should not be at the detriment of their sensory and physico-chemical attributes.

African yam bean has been used by many to complement, supplement, fortify and enrich several staples such as breakfast meals (Babarinde et al., 2019) biscuits (Idowu, 2014; Igbabul et al., 2015; Okoye and Obi, 2017) and traditional snacks such as *Kokoro* (Idowu, 2015b), instant noodles (Effiong et al., 2018), cereal blends (Okoye et al., 2017) among others. Most authors have erroneously used the term fortification and enrichment in their use of AYB to improve and enhance the nutritional qualities of food. Great attention must be given to the use of these terms so that future researchers are not left confused. The richness of the bean in protein makes them a good fortifying candidate for different foods. Hence, their use in food fortification and enrichment can improve food security and human nutrition.

### 4.1 Snacks and cookies

Snacks and cookies are widely consumed all around the world by people from different socioeconomic status, this is because they are already processed and packaged as a ready-to-eat snack (Adeola and Ohizua, 2018). Oyeyinka and Oyeyinka (2018) stated that snacks are good carriers for nutrients for selected consumer groups. Generally, snacks produced from cereal flours are usually deficient in protein and essential amino acids. AYB has been used in different studies to fortify snacks and cookies (Table 4). Idowu (2014) used AYB as a flour composite to improve the protein content of biscuits. The inclusion of up to 40% AYB flour resulted in significant increase in protein up to 53% and ash up to 77% of the new product (Idowu, 2014). The increased ash content indicates the presence of certain minerals in AYB that were originally absent in the control sample. Sensory test indicates overall acceptability of biscuits made from AYB at all levels and compares favourably with the control. We recommend the use of a larger sensory panel (trained and untrained) across all age groups to ascertain the acceptability among a larger consumer population.

**Table 4:** Proximate composition of African Yam Bean enriched, fortified composite and complemented foods (%).

Product	Ash	Protein	CHO	Fibre	Fat	Moisture	References
Biscuit from wheat-AYB composite flour	1.37–2.42	9.61–14.71	NR	NR	12.53–8.13	11.04–11.24	(Idowu, 2014)
Cookies from composite flour of wheat, cocoa-yam and AYB	2.40–2.64	10.44–14.73	71.84–63.94	1.63–2.42	3.01–6.73	10.68–8.54	(Igbabul et al., 2015)
Kokoro made from Maize-AYB flour blend	1.87–2.42	9.91–13.11	NR	NR	21.08–34.25	1.40–2.13	(Idowu, 2015b)
Breakfast meal from sweet potato – AYB mixes	2.33–2.84	4.25–8.83	74.99–78.80	4.63–3.35	0.45–0.84	10.49–8.79	(Babarinde et al., 2019)
Enriched *ogi* from Maize – AYB flour composite	0.59–0.73	0.02–1.07	10.65–9.80	0.07	0.06–0.05	85.81–86.42	(Ukegbu et al., 2015)
Enriched *Agidi* from Maize – AYB flour composite	1.54–1.65	2.20–2.74	11.32–10.34	0.45–0.64	4.28–4.92	18.53–32.30	(Ukegbu et al., 2015)
Composite flour from Wheat – OFSP - AYB	0.57–1.39	11.61–12.85	70.35–69.23	2.03–3.16	1.41–1.96	13.95–11.40	(Effiong et al., 2018)
Instant noodles from Wheat OFSP composite flour fortified with AYB	1.35–2.11	11.67–13.04	70.90–67.90	2.13–3.38	1.70–2.19	13.24–11.53	(Effiong et al., 2018)
Complementary food from sorghum, crayfish and AYB	2.77–3.67	13.56–23.88	72.36–60.34	1.46–2.15	1.85–3.64	9.46–10.33	(Egbujie and Okoye, 2019)
*Sorghum bicolor*-AYB weaning food	3.30–5.81	10.15–15.40	70.77–55.08	2.30–1.13	9.00–6.19	2.50–8.14	(Ijarotimi and Bakere, 2006)
Fufu fortified with AYB	NR	4.9–7.8	NR	NR	NR	NR	(Aniedu and Aniedu, 2014)
Complimentary diet from maize, AYB and pigeon pea	4.26–3.74	10.52–24.29	73.02–58.49	3.17–3.66	1.46–1.85	7.05–7.65	(Oludumila and Enujiugha, 2017)
Amala from Plantain –AYB blend	4.01–4.75	14.43–18.24	62.35–57.61	6.78–7.83	1.71–3.56	9.97–9.57	(Akinsola et al., 2018)
Maize – AYB Blend for *tuwo*	1.39–2.73	9.61–22.73	NR	1.34–5.81	4.53–2.66	NR	(Idowu, 2015a)

NR: Not reported.

Igbabul et al. (2015) also used AYB flour and fermented cocoyam (CCY) flour composite at different concentrations (w/w) for cookies production. According to their report, there was a marginal increase in protein and ash content in the newly developed product. A significant (*P* < 0.05) increase in protein of about 38% was noticed when up to 40% AYB was included (Igbabul et al., 2015). An increase in ash and fibre content was also reported in the same study. The increased fibre is expected as fibre content in cocoyam added to the low fibre present in the wheat and AYB flour. Sensory score shows the acceptability of all samples with no significant difference noticed, however, the control sample was rated best for all sensory parameters.

African yam bean has also been used as an ingredient to enhance the proximate and nutritional component of *Kokoro*. *Kokoro* is a traditional snack consumed in South Western Nigeria (Abegunde et al., 2014; Idowu, 2015a, Idowu, 2015b). It is usually prepared from maize flour which is deficient in most nutrients and rich in carbohydrate (Abegunde et al., 2014). Idowu, (2015b) developed *kokoro* from maize-AYB paste, a significant increase, in protein was reported. There was approximately 32% increase in protein with the inclusion of up to 40% AYB (Idowu, 2015b). The mineral component of Kokoro also increased significantly with an increase in AYB addition which makes AYB enriched Kokoro a source of important minerals particularly among households and communities prone to micronutrient malnutrition. The following minerals were reportedly present; calcium, sodium, zinc, magnesium, iron, copper, manganese and potassium. All developed products were acceptable to untrained sensory panelists.

### 4.2 Breakfast, weaning and instant diets

Breakfast and weaning diets for infants are commonly produced from cereals, most of which are deficient in the most important nutrients for healthy living. The development of breakfast cereals using various protein-rich foods as ingredients has previously been reported by different authors (Ijarotimi and Bakere, 2006; Onweluzo and Nnamuchi, 2009). The use of AYB in the development of different nutritionally – improved breakfast and weaning diets has also been extensively studied (Table 4). More recent is the addition of AYB to sweet potato in developing a breakfast diet by Babarinde et al. (2019). The results from this study indicated a significant (*P* < 0.05) increase in protein with over a 100% increase recorded. The inclusion of AYB at 15% also significantly improved the swelling capacity of the new product. Sensory scores show that the control sample and 15% AYB flour inclusion were the most acceptable. The inclusion of up to 30% AYB flour resulted in a poor rating of the product in terms of colour, aroma and taste due to a noticeable beany taste in the product. This could be eliminated by choosing appropriate processing methods for the flour used. The enhancement of this breakfast diet shows the importance of AYB as an ingredient in the production of protein-rich diets for infants.

Effiong et al. (2018) developed instant noodles with wheat, Orange fleshed sweet potato (OFSP) and AYB flour. The authors reported approximately 10% increase in protein, a 42% increase in ash, and a 37% increase in fibre content of the noodles. The fat content of the new product increased slightly. This may confer additional taste to the noodles, as the presence of fat is likely to enhance tastiness (Bolarinwa et al., 2019) and hence improve consumer acceptance. A marginal increase in vitamin A content of the new product was reported due to the inclusion of OFSP (Effiong et al., 2018). A diet high in vitamin A is good for eyesight and immune system functions. Sensory analysis indicated general acceptance of the new product. Although, we recommend that a large sensory panel with a wide demographic variation that mirrors the opinion of potential consumers of the newly developed food product is used.

### 4.3 Complementary foods

Complementary diets have been developed using AYB. AYB alongside crayfish (CF) was used in the preparation of a composite with sorghum (Egbujie and Okoye, 2019). A huge (about 76%) and a significant increase in protein content were reported. A slight but significant increase in the ash and fibre content was noticed. There was also a noticeable vitamin (niacin, ascorbic acid, thiamine, Vitamin A) increase with increasing AYB and CF. A protein-rich diet can help ameliorate the problem of protein-energy malnutrition (Arise et al., 2015; Ijarotimi and Bakere, 2006; Oyeyinka and Oyeyinka, 2018).

In another study by Ijarotimi and Bakere (2006), AYB was used in the development of a complementary diet with *Sorghum bicolor* which was touted as an inexpensive weaning food for infants. Generally, most cereal-based diets are deficient in important micronutrients and protein. In this study, the authors reported a marginal increase in protein composition for all complemented samples (Table 4). Generally, enhanced protein content values are reported for fortified and enriched cereals (Mbata et al., 2009; Abioye, 2015; Arise et al., 2015; Afolabi et al., 2018; Oyeyinka and Oyeyinka, 2018; Adeyemo and Olufemi, 2019; Nwadi et al., 2020). Fortification results in improved mineral content of the new product. The authors recommended the use of 20% AYB for use in infant weaning foods as this combines high nutritional content with tolerable levels of anti-nutrient composition for infants (Ijarotimi and Bakere, 2006). Sensory perception of consumers was not reported for this study and as such its full acceptability may not be ascertained.

Oludumila and Enujiugha (2017) also developed *ogi* from a composite of maize – AYB – pigeon pea. The need for this addition is essential because pure maize flour *ogi* is biologically deficient in important nutrients for the healthy development of infant children (Arise et al., 2015). The addition of up to 15% AYB resulted in a new product with superior protein content (over 100% increase) (Oludumila and Enujiugha, 2017). The complimentary samples show better nutritional quality than 100% maize flour *ogi*. The mineral composition of the new product also improved massively (Oludumila and Enujiugha, 2017). The huge increase in protein cannot only be attributed to the use of AYB but also the inclusion of pigeon pea, that contains a comparable protein profile to AYB. The acceptability index among consumers was not reported in this study and as such consumer perception and acceptability of the product cannot be ascertained.

### 4.4 Traditional foods

Traditional foods are generally prepared and consumed among people who live in rural areas and occasionally by urban dwellers (Nwadi et al., 2020). Many of these foods are prepared from carbohydrate-dense ingredients with low protein content. African yam bean just like other protein-rich foods such as Bambara groundnut and soybean has been used as an ingredient in the preparation of traditional foods. AYB has been used to enrich *fufu* flour (Aniedu and Aniedu, 2014), a fermented product of cassava. Samples were classified as treated (toasted and fermented) and untreated. A general increase in protein in all fortified samples was reported. Treatment of AYB did not affect the protein content of the fortified *fufu* flour. Because of its enhanced protein content, AYB enriched *fufu* can be consumed among those who cannot get animal protein from meat and fish without a sense of eating a diet deficient in protein. Sensory studies show wide acceptability of the enriched *fufu* as overall acceptability compared favourably with the control sample, although the *fufu* sample enriched with 5% AYB was rated best. Consumer perception of the acceptability of AYB enriched *fufu* may not be reliable as discussed in this study because the use of a 20 man panel may not mirror the perception of a larger consumer group. Oyeyinka and Oyeyinka (2018) earlier suggested the importance of a larger panel as it demonstrates the reliability of consumer perception data in literature.

In another study by Ukegbu et al. (2015), AYB was used to enrich *ogi* and *agidi* (maize gruels) commonly consumed by adults and used in weaning babies among the south-western populace in Nigeria. Nutritionally, *ogi* and *agidi* are poor and do not offer sufficient amounts of the nutrients required for growth (Arise et al., 2015). Additionally, these authors stated that children weaned solely with *ogi* suffer from malnutrition, therefore the need for fortification and enrichment of weaning foods become necessary and hence needed to be fortified. *Ogi* is usually prepared by steeping maize grain in water for approximately 72 h during which fermentation occurs (Oyeyinka and Oyeyinka, 2018), after which they are wet milled and sieved using a muslin cloth to separate the bran and hull (Abioye, 2015; Ukegbu et al., 2015). Results from the prepared *ogi* and *agidi* with AYB shows a significant (*P* < 0.05) increase in protein (Ukegbu et al., 2015). The protein content of the enriched *ogi* and *agidi* were superior to the unenriched samples. Sensory acceptance of AYB - enriched *ogi* and *agidi* was observed. The colour of the fortified product was rated poorly. The use of crayfish with AYB in the production of *ogi* may create challenges in understanding the exact effect of AYB on the protein content of the new product. As stated before, the use of a proper mixture design may be useful in understanding the effects of each ingredient on the protein content of newly developed product.

*Amala*, a traditional food commonly consumed in South-western Nigeria was also complimented with AYB. It is usually prepared from yam flour, cassava flour (*lafun*) and plantain flour (*Amala ogede*). Akinsola et al. (2018) reported significant increase (*P* < 0.05) in protein content. The addition of 25% AYB resulted in approximately 27% increase in protein value. There was an increase in fibre content of the developed product. Increased fibre content is important as fibre aids distention and motility during digestion (Akinsola et al., 2018). The 20% AYB enriched plantain was deemed best of all samples. The addition of AYB influenced the functional property of the resulting product. For instance, a slight but insignificant increase was recorded for swelling capacity. Swelling capacity has a direct effect on stiff dough consistency. This product is recommended for those diagnosed with diabetes and cardiovascular disorder for effective management.

Another traditional food that has been developed using AYB blend is *Tuwo, Tuwo* is a traditional non-fermented food prepared from maize and popularly consumed in the Northern part of Nigeria (Bolade and Adeyemi, 2012). Because of the deficiency of important nutrients in meals prepared from maize (Arise et al., 2015; Idowu, 2015a). Idowu, (2015a) included AYB in the preparation of *Tuwo*. There was increase in the protein content of the enriched product, *Tuwo* enriched with AYB up to 40% resulted in approximately 137% increase. A marginal increase in ash and fibre content of the *Tuwo* was also reported (Idowu, 2015a). An increase in mineral content was also observed. The author reported that *Tuwo* prepared from 20% AYB was best in terms of the sensory attribute with the consumer panel. The reliability of the acceptability index of all samples enriched with AYB needs further validation with a larger panel size. This further may be recommended for consumption among all age groups for health and vitality.

## 5 Processing of African yam bean and its effect

Most legumes are not fit for consumption in their raw state except they undergo some processing operations. Processing is done to improve the quality and functionality of foods, for increased bioavailability of nutrients and reduction of undesirable compounds such as toxins and non-nutritional compounds. African yam bean is one of such legumes that requires careful and appropriate processing for consumer palatability and safety. This is because pulses are generally high in compounds that alter normal metabolic processes. Over the years, the bean has been pre-processed and processed using different methods. Such methods includes: germination, roasting, fermentation, soaking as well as cooking or boiling.

Germination in food processing is a process where seeds are steeped or soaked for a period of time and then sprouted at the early stage of development of seeds to plant. Some authors have reported that germination results in improved flavour, nutritional composition, amino acid content and reduction of non-nutritional compounds in many legumes (Enujiugha et al., 2003; Rumiyati et al., 2012). In addition to this, germination results in breakdown of complex carbohydrates in legumes into simple sugars through the action of endogenous enzymes thereby enhancing digestibility (Nkhata et al., 2018). It was reported that germination of AYB resulted in improved protein quality and enhanced the bioavailability and digestibility of other nutrients with a marked decrease in non-nutritional compounds like oxalate, phytic acid, trypsin inhibitors amongst others (Nwosu, 2013). Germination also improved the antioxidant content of AYB, for instance, it has been demonstrated from literature that the antioxidant content and free radical scavenging activity of the bean improved significantly after germination (Uchegbu, 2015). Hence, germinated AYB meal is said to be a good dietary inclusion for individuals with complications from oxidative stress, hyperlipidemia, and diabetes.

Roasting has also been used in the processing of AYB and was reported to have increased the minerals composition such as calcium, potassium, phosphorus, magnesium, etc of the bean as well as resulting in great decrease in non-nutritional compounds (Ndidi et al., 2014). Ene-Obong and Obizoba (1996) stated that roasting improved the phosphorus content of the bean, while tannin content was non-detectable, although this was attributed to the removal of the seed coat that resulted from roasting. This method has been described as a viable method of preparing AYB snacks which are mostly consumed with palm kernel in the eastern part of Nigeria (Ene-Obong and Obizoba, 1996).

Fermentation is used in the modification of the biochemical composition of foods through the action of enzymes and bacteria (Nkhata et al., 2018). This process improves the functional and organoleptic properties of many legumes as well as their bioavailability. It has been reported that fermentation led to a significant (*P* < 0.05) decrease in phytate composition and an increase in the phosphorus content caused by the breakdown of phytates by the activity of the enzyme phytase (Ene-Obong and Obizoba, 1996).

Soaking has been used in the pre-processing of AYB. It involves the hydration of legumes for a specified period before cooking or further processing to soften the seed thereby reducing the cooking time. For instance, Ene-Obong and Obizoba (1996) reported that soaking of AYB for 6–12 h before cooking reduced the cooking time of the bean by over 50%. In a similar study on Bambara groundnut, the hard-to-cook attribute of the seed was alleviated by soaking before cooking at elevated temperatures, which led to a significant decrease in cooking time (Ojo, 2018). Although this processing operation seems desirable, it may result in loss of important minerals. For instance, it has been reported that soaking for up to 24 h resulted in a significant decrease in calcium, magnesium, and iron content of AYB as these minerals leached into soaking water (Ene-Obong and Obizoba, 1996). A slight increase in protein up to 10% and a minute increase in phosphorus was observed by this author. In all, the processing of AYB is aimed at improving the nutritional, functional and organoleptic quality of the bean while decreasing the non-nutritional compounds present.

## 6 Limitations to the use of African yam bean

Over the years, there have been limitations to the use of many African legumes, due to certain attributes that have been associated with them. The two most important limitations that have been discussed by different authors for many years are their hard-to-cook nature (Ojo and Ade-omowaye, 2015; Duodu and Apea-Bah, 2017; Maphosa and Jideani, 2017) and the presence of non-nutritional compounds otherwise known as anti-nutrients (Prasad and Singh, 2015; Ajibola and Olapade, 2016; Duodu and Apea-Bah, 2017). African yam bean is one of many leguminous crops that exhibit these limitations. The crop requires an extended cooking time because of their hard-to-cook characteristic which has restricted their use and processing among the consuming populace and households (Aminigo et al., 2009; Idowu, 2014; Ojo & Ade-omowaye, 2015). Prolonged cooking time is cost-intensive, results in high energy consumption, loss of some important micronutrients that may have leached as a result of an extended cooking period which may damage important nutrients. Although extended cooking contributes to the reduction of non-nutritional compounds present in the bean. The hard-to-cook condition of many pulses like AYB can be minimized by soaking prior to hydrothermal treatments (Ojo, 2018). This author reported that hydration of hard-to-cook pulses prior to boiling is an effective way of reducing cooking time and energy consumption. Nwosu (2013) stated that malting and germination of AYB before cooking may also reduce the prolonged cooking time of this bean.

In addition to prolonged cooking period, the presence of non-nutritional compounds in AYB has also been a limiting factor for its use (Oboh et al., 1998; Coelho et al., 2007; Ajibola and Olapade, 2016). Non-nutritional compounds are naturally occurring chemical compounds in plants with the potential to alter the absorption of nutrients and slow down metabolic processes (Oboh et al., 1998). These compounds may interrupt the bioavailability of mineral compounds like calcium and iron if appropriate processing techniques are not followed (Ghavidel and Prakash, 2007). The non-nutritional compounds that have been reported to be present in AYB include; lectin, tannins, phytic acid, oxalate, saponins, stachyose, alkaloid, raffinose, trypsin inhibitors, hydrogen cyanide (Nwosu, 2013; Ajibola and Olapade, 2016; Duodu and Apea-Bah, 2017; Maphosa and Jideani, 2017). Some authors have suggested certain processing methods for the reduction of these compounds, among them are; soaking, malting, and germination (Nwosu, 2013; Duodu and Apea-Bah, 2017), boiling and roasting (Aremu et al., 2016), irradiation and fermentation (Duodu and Apea-Bah, 2017), dehulling, boiling and steaming (Maphosa and Jideani, 2017). The soaking of AYB for 24 h prior to cooking has been reported to be a viable way of reducing non-nutritional compounds (Nwosu, 2013). This author also reported that germination and malting reduce the non-nutritional contents. The use of these methods has also been reported in the reduction of non-nutritional compounds present in other similar pulses (Vidal-Valverde et al., 1994). Aside from extended and rigorous cooking conditions and non-nutritional compounds, Maphosa and Jideani (2017) also stated that the limited use of AYB may be attributed to poor yield in certain areas, low seed availability, rigorous labour needs at maturity, lack of convenient food applications and low level of awareness, as well as bloating and flatulence.

## 7 Food security; making the best of African yam bean

The 2002 report by the Food and Agricultural Organisation (FAO) described food security as a scenario where everyone at all times possesses social and economic access to nutritious, safe and adequate quantities of food that can meet their dietary requirements for a good and healthy life. African yam bean as earlier discussed is a readily available food crop in western and eastern Africa that is still largely underused despite its nutrient density that is capable of contributing to the consumer's daily nutrient reference values (NRV). Pregnant women, nursing mothers and their children still face the risk of undernutrition and malnutrition in several countries of the world, particularly in Africa. This is because they live on diets that do not meet the NRV for growth and healthy living. There are current projections that acute and severe food insecurity may plunge many parts of Africa and war-ravaged countries in the middle east due to climate change and economic instability which may affect over 30 million people (FSIN, 2019). This can be diminished by giving attention to underutilized crops in many of these countries, first in addressing the problem of hunger and then supplying nutrients needed for growth and health. African yam bean is one of such crop that is capable of helping to mitigate food insecurity and malnutrition. Their nutrients are comparable to other commonly consumed legumes and can serve as a replacement for animal proteins in human diet (Abioye et al., 2015; Igbabul et al., 2015; Akinsola et al., 2018; Agunwah et al., 2019).

## 8 Conclusion and future perspective

This review has detailed the usefulness of African yam bean, an underexploited leguminous plant in west Africa. AYB 's use as a food fortifier and enricher, as well as its use in complementing foods that are poor and deficient in important nutrients, were also discussed. Although this crop has numerous potentials, they are yet to be fully harnessed. With the possible negative impact of climate change on food crops and by extension food security, it has become imperative to remember these almost forgotten crops in helping to mitigate these negative impacts.

It is recommended that more awareness about the crop be embarked upon by extension workers and government bodies saddled with food security and sustainable development. Additionally, awareness should also focus on the deliberate commercialisation of the crop and increase its use among food manufacturers. The development of AYB meat analogue should also be considered. Sensory acceptability of enriched, complemented, and fortified diets prepared from AYB can be enhanced using experimental designs. This is crucial to determining the optimum quantities to use in the preparation of these foods on a commercial and industrial scale. The use of market sensory survey in determining and ascertaining broad consumer perception should be done.

Furthermore, the presence of phytochemicals and antioxidant compounds, slowly digestible starch and fibre in AYB makes them a viable food crop in the development of functional foods. Phytochemical extracts from AYB can be further developed into micro and nano capsules for inclusion in foods. The full profile can be determined using the LC-MS and NMR. *In vitro* and *In vivo* digestibility studies can be embarked upon to determine the bioavailability of the nutrients and antioxidant contents, in enriched and fortified foods developed from AYB.

## Declarations

### Author contribution statement

All authors listed have significantly contributed to the development and the writing of this article.

### Funding statement

This work was supported by the South African National Research Foundation (NRF) under the Thuthuka grant (TTK180424323899).

### Competing interest statement

The authors declare no conflict of interest.

### Additional information

No additional information is available for this paper.
